# The Effect of a Home-Based Tele-Exercise Training Program on the Quality of Life and Physical Performance in Breast Cancer Survivors

**DOI:** 10.3390/sports11050102

**Published:** 2023-05-10

**Authors:** Andreana Andrioti, Argyro Papadopetraki, Maria Maridaki, Anastassios Philippou

**Affiliations:** 1 Medical School, National and Kapodistrian University of Athens, 115 27 Athens, Greece; andreand@med.uoa.gr (A.A.); argpapa@med.uoa.gr (A.P.); 2 Faculty of Physical Education and Sport Science, National and Kapodistrian University of Athens, 172 37 Athens, Greece; mmarida@phed.uoa.gr

**Keywords:** breast cancer, survivors, exercise, tele-exercise, fatigue, anxiety, quality of life, post-traumatic stress disorder (PTSD)

## Abstract

The number of breast cancer (BCa) survivors has been steadily increasing due to advances in anti-cancer treatments, though these individuals suffer from various cancer- and treatment-related long-term side effects. The present study aimed at investigating the effects of a home-based tele-exercise training intervention on physical- and mental health-associated parameters in BCa survivors. A total of 13 female BCa survivors (age: 58.31 ± 3.13 years, BMI: 25.68 ± 0.62 kg/m^2^, waist circumference: 96.54 ± 1.84 cm) participated in a two-month group tele-exercise program twice per week which included aerobic, resistance and flexibility exercises. The results of the study revealed that the tele-exercise intervention improved participants’ body mass index (BMI) (*p* < 0.001), waist circumference (*p* < 0.001), cardiorespiratory fitness (6 min walk test) (*p* < 0.001) and muscle function (sit to stand (*p* < 0.01), sit ups (*p* < 0.001) and push-ups (*p* < 0.001)). Beneficial effects were also observed on perceived anxiety (Zung Self-Rating Anxiety Scale) (*p* < 0.001), post-traumatic stress disorder (PTSD) symptoms (PCL-C) (*p* < 0.01), self-reported fatigue (*p* < 0.001), quality of life (QoL) (*p* < 0.05) and physical (*p* < 0.05), cognitive (*p* < 0.01) and emotional (*p* < 0.05) functioning (EORTQ-QLQ-C30). Our findings suggest that common cancer- and treatment-related adverse effects on physical performance, mental health and the overall QoL can be ameliorated through tele-exercise training programs in BCa survivors.

## 1. Introduction

Breast cancer (BCa) is the most common type of cancer in females, and it is estimated that 1 in 8 women will be diagnosed with breast cancer at least once during their lifetime [1,2,3]. Interestingly, about 1/3 of BCa cases could be prevented by modifying well-established risk factors, such as reduced physical activity levels, obesity and excess alcohol consumption [4]. However, despite the high incidence rates which are partially attributed to frequent screening, the number of BCa survivors has been steadily increasing due to the advances in anti-cancer therapies [5]. Indeed, the five-year survival rate in females reaches 99% for stage I BCa, while for stages II, III and IV, the survival rate is 93%, 75% and 29%, respectively, for all ages and ethnicity groups [2]. 

It is noteworthy that the BCa diagnosis appears to have a long-term impact on survivors’ daily lives by affecting them both physically and emotionally and, thus, compromising their quality of life (QoL). Poor cardiometabolic health, sarcopenia, osteoporosis, reduced range of motion, chronic fatigue and pain are some of the adverse effects of common anti-cancer therapeutic strategies, including surgery, chemotherapy, radiation and hormonal therapy [6,7]. Additionally, cancer survivors, especially those who were subjected to intensive treatments, often suffer from cognitive impairment, depression, anxiety, post-traumatic stress disorder (PTSD) and a persisting fear of disease recurrence [8,9]. 

However, over the last few years research evidence supports that health-promoting lifestyle interventions in breast cancer survivors have multiple positive effects on physiological and psychometric parameters [10,11]. In particular, physical exercise offers beneficial effects on cardiorespiratory health, muscle function, weight control and fatigue, since it induces acute and chronic adaptations in the physiological systems [12,13,14]. More specifically, some of the abovementioned exercise-induced adaptations include the compensation of bone and mass loss, increase of antioxidant capacity, regulation of sex hormones, increased insulin sensitivity and enhanced immune response [13,15]. In addition, exercise interventions counteract many psychological and emotional distresses, being effective in the reduction of anxiety, PTSD and depression, as well as in enhancing the overall health-related QoL [16,17,18]. The exercise-induced physiological mechanisms implicated in the modulation of stress-related disorders include the increased reactivity of hypothalamic–pituitary–adrenal (HPA) axis, neurogenesis, secretion of myokines and monoamine and opioid systems. Moreover, exercise reduces stress and anxiety levels by helping individuals expose themselves to and be familiarized with stimuli and conditions previously perceived as threatening, while it also improves self-efficacy and serves as a distraction to stressful events [19]. Nevertheless, a considerable number of breast cancer survivors does not engage in structured physical activity or exercise programs, either because they are unaware of the exercise-induced health benefits or because exercise training facilities are not easily accessible [20]. Home-based exercise though, utilizing advanced technology, can be used to overcome distance barriers and enable a greater number of survivors to participate in physical activity programs [21]. Indeed, at the onset of COVID-19 pandemic, many people turned towards home-based exercise training programs, performed either under remote supervision or through social media videos in order to counteract physical inactivity due to restrictions of outdoor mobility and participation in essential out-of-home activities [22,23,24]. Consequently, home-based exercise, utilizing digital technologies, has been increasingly used in oncology patients as growing evidence indicates that it is a feasible, safe and effective intervention for them [25]. Indeed, remotely performed exercise training programs, especially in breast cancer survivors, have been shown to be an accessible and efficient exercise intervention which improved their physical performance, body composition, fatigue and overall quality of life [22,26].

Although tele-exercise is gaining momentum, available data on supervised distance exercise programs in BCa survivors is still limited and further research is needed to verify the physical and mental health benefits of tele-exercise to these patients. Thus, the aim of the present study was to determine the potential effects of a two-month home-based supervised tele-exercise program, containing both aerobic and resistance training, on physical performance, psychological parameters and QoL in BCa survivors.

## 2. Materials and Methods

### 2.1. Consent Form and Ethical Approval 

All participants, who voluntarily enrolled in the present longitudinal intervention study, were thoroughly informed about the experimental procedures and signed a written informed consent form that was approved by the Ethics Committee of the Medical School of the National and Kapodistrian University of Athens. All privacy law regulations were followed regarding data collection and handling.

### 2.2. Study Participants

A total of 13 women BCa survivors, aged 58.31 ± 3.13 years, volunteered to participate in the study after the approval by their personal physician. The participants enrolled in the study according to the following inclusion criteria: they should have been diagnosed with breast cancer within the last 5 years (the average time after the completion of their last treatment was 2.94 ± 0.32 years), have no distant metastasis, have undergone mastectomy, and have completed chemotherapy and/or radiation therapy. In addition, they had to be free of severe cardiovascular, renal, pulmonary or psychiatric disease and to be able to understand and speak Greek. 

### 2.3. Tele-Exercise Training Program

BCa survivors participated in a home-based group tele-exercise training program consisting of two sessions per week with each session lasting one hour and containing both aerobic and resistance exercises (Table 1). The program was implemented under the synchronous supervision of an exercise scientist through video-calls, during which all exercises were explained in detail and individual corrective feedback was provided to each participant in every session.

### 2.4. Data Collection

The tele-exercise training program lasted 2 months and re-assessment of physical performance and somatometric characteristics was carried out every 4 weeks. Moreover, participants self-evaluated their quality of life and perceived post-traumatic stress and anxiety at the beginning and after the completion of the training program. 

#### 2.4.1. Somatometric Characteristics

Participants were asked to stand barefoot with their backs against a height rod in order to measure their height, while an electronic precision balance was used to assess their body mass. Afterward, the body mass index (BMI) was determined [BMI (kg/m^2^) = body mass (kg)/body height^2^ (m^2^). According to the World Health Organization (WHO), a BMI ranging from 18.5 kg/m^2^ to 24.9 kg/m^2^ represents a healthy body weight range, whereas if it exceeds the 25 kg/m^2^ or 30 kg/m^2^, the individual is considered overweight or obese, respectively [27]. In addition, the participants’ waist circumference was measured in the middle of the distance between the iliac crest and the last palpable rib using a measuring tape [28].

#### 2.4.2. Cardiorespiratory Fitness

The 6 min walk test (6MWT) was utilized to assess BCa survivors’ aerobic capacity [29]. Patients were instructed to walk at their own pace, aiming to cover as much distance as possible, over a 6 min duration, while they were allowed to stop and rest if necessary. The test was performed on a horizontal, flat and hard surface and the walked distance was recorded. Heart rate at rest and after the completion of the 6MWT was measured, while the 20-point Borg scale was used to evaluate dyspnea and leg fatigue. Relative contraindications were set so as not to participate in the assessment if resting heart rate (HR) was higher than 100 bpm, Systolic Blood Pressure (SBP) was higher than 145 mmHg and Diastolic Blood Pressure (DBP) was higher than 95 mmHg [30]. 

#### 2.4.3. Muscle Function

Participants’ muscle function of various muscle groups of lower and upper extremities as well as of the abdominals was assessed in the BCa survivors before, during and after the completion of the tele-exercise training program, using three different muscle function assessment tests. Specifically, the “sit-to-stand test”, which measures how many times an individual with the arms crossed in front of the chest can sit and rise from a chair in 60 s, was used to evaluate muscle function of lower extremities [31]. An armless chair was used, and instructions were given to participants to execute the movements as fast as possible. Similarly, the muscle function of upper extremities and the abdominals was assessed by measuring the number of repetitions of “push-ups” and “sit-ups” performed in 60 s, respectively.

#### 2.4.4. Quality of Life

Quality of life was self-reported by the BCa survivors, using the European Organization for Research and Treatment of Cancer Quality of Life Questionnaire Core 30 (EORTC QLQ-C30, version 3.0), a widely used tool for the assessment of perceived QoL in cancer patients [32]. This instrument incorporates both single-question answers and multi-item scales, including cancer-specific symptoms, overall health status and physical, cognitive, emotional, social and role functioning. The total score generated on each scale ranges from 0 to 100 and the higher the score, the greater the response level on the particular scale. Thus, high scores in symptom scales indicate a high level of cancer-related symptomatology, while reversely, high scores in functioning represent superior health status and well-being. 

#### 2.4.5. Post-Traumatic Stress

The Post-Traumatic Stress Disorder (PTSD) Checklist-Civilian version (PCL-C) was used to evaluate typical PTSD symptoms. It is composed of 17 self-reported questions based on the fourth revision of Diagnostic and Statistical Manual of Mental Disorders (DSM-IV) diagnostic criteria regarding PTSD. BCa survivors were instructed to answer carefully to what extent they experienced each symptom within the last month, rating the level of symptoms from one (not at all) to five (extremely). The total score represents the extent of PTSD symptomatology with the severity increasing as the total score rises. In general, a score greater than or equal to 50 is considered to reflect a full PTSD diagnosis [33]. 

#### 2.4.6. Anxiety

Anxiety was evaluated using the Zung Self-Rating Anxiety Scale (SAS) which is a 20-item tool with psychometric properties. Respondents should evaluate each item on a 4-point scale, grading from “rarely” to “always”, according to their individual perceived situation in the past week. In order to reduce the possibility of bias in the responses, while 15 items of the questionnaire contain negative claims regarding anxiety, the remaining five items convey positive statements. The total anxiety index is obtained by multiplying the sum of the score in each item by 1.25. Thus, the raw score ranges from 20 to 80; however, the final anxiety index lies between 25 and 100. Similar to the PCL-C assessment tool, a low index in the Zung scale signifies poor anxiety-related manifestations [34].

### 2.5. Statistical Analysis

Statistical analysis was performed using the Graphpad Prism statistical package (GraphPad Software, Version 5.03, Inc., San Diego, CA, USA). Mean and standard error of the mean (ΜΕAΝ ± SE) were used for descriptive statistics and Shapiro–Wilk test was performed to assess the normality of data distribution. A parametric paired *t*-test or a non-parametric Wilcoxon sing-ranked test was employed for the analysis of questionnaires’ scores at the two time points of evaluation depending on the normal or non-canonical distribution of the data, respectively. One-way analysis of variance (ANOVA) or the non-parametric alternative of Friedman test was utilized to examine the potential differences in the physiological and functional parameters before, during and after the intervention. No missing values occurred and the statistical significance was set at *p* < 0.05.

## 3. Results

### 3.1. Somatometric Characteristics

**Figure 1:**
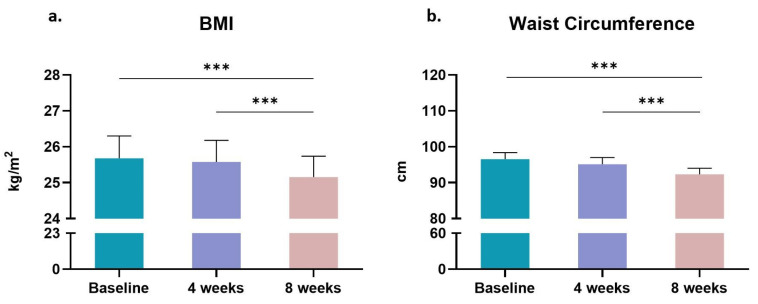
Changes in (**a**) body mass index (BMI) and (**b**) waist circumference in women breast cancer survivors participated in the 8-week tele-exercise training program. Data are presented as mean ± SE. Significantly different ***: *p* < 0.001.

The somatometric characteristics of the BCa survivors that participated in the present study are shown in Table 2. The 8-week tele-exercise training program decreased their BMI compared to baseline values (*p* < 0.001), while no differences (*p* > 0.05) were found four weeks after the initiation of the training program (Table 2 and Figure 1a). Similarly, significant reductions were found in waist circumference after the completion of the eight-week exercise intervention (*p* < 0.001; Table 2 and Figure 1b).

### 3.2. Cardiorespiratory Fitness

**Figure 2:**
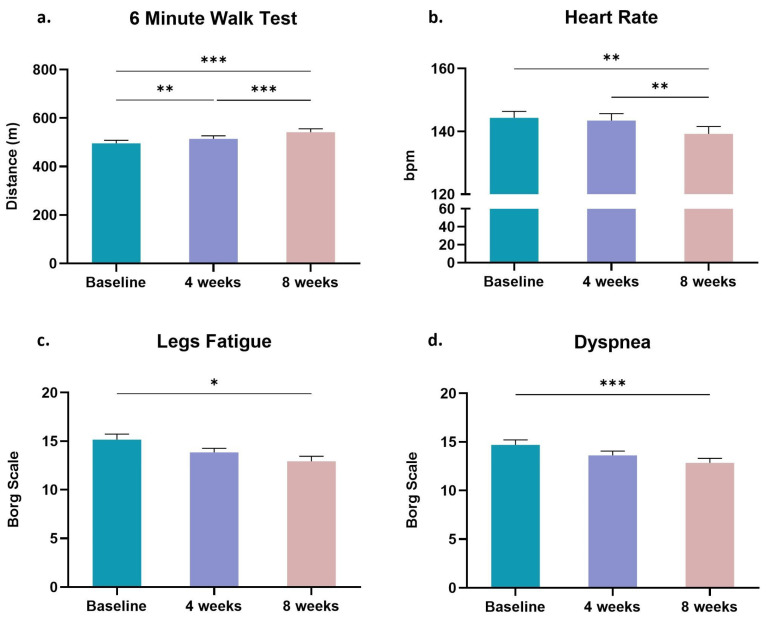
Changes in cardiorespiratory fitness-related parameters as a result of the tele-exercise training program in the BCa survivors: (**a**) walked distance in 6MWT, (**b**) heart rate at the completion of the 6MWT, and perceived (**c**) leg fatigue and (**d**) dyspnea at the completion of the 6MWT. Data are shown as mean ± SE. Significantly different *: *p* < 0.05; **: *p* < 0.01; ***: *p* < 0.001. bpm: beats per minute.

Changes in the evaluated cardiorespiratory fitness-related parameters of the participants because of the tele-exercise training program are presented in Figure 2. Specifically, the walked distance in the 6MWT increased from 495.38 ± 13.09 m at baseline to 514.62 ± 12.49 m after four weeks of the exercise training (*p* < 0.01) and to 541.54 ± 14.00 m after the completion of the eight-week training program (*p* < 0.001; Figure 2a). Moreover, the HR at which they finished this submaximal field test decreased significantly only after the eight-week training period compared to baseline (144.31 ± 2.10 vs. 139.23 ± 2.31 bpm; *p* < 0.01; Figure 2b). Similarly, significant improvements were reported after the eight-week exercise training program regarding leg fatigue (baseline: 15.15 ± 0.58 vs. eight-weeks: 12.92 ± 0.52; *p* < 0.05; Figure 2c) and dyspnea (baseline: 14.69 ± 0.51 vs. eight-weeks: 12.85 ± 0.46; *p* < 0.001; Figure 2d) perceived by the participants at the completion of the 6MWT. No significant effect of the exercise training on the resting HR was found (69.46 ± 1.10 bpm at baseline vs. 69.31 ± 1.06 bpm at the fourth week vs. 67.08 ± 0.76 at the eighth week of the tele-exercise training program; *p* > 0.05). 

### 3.3. Muscle Function

**Figure 3:**
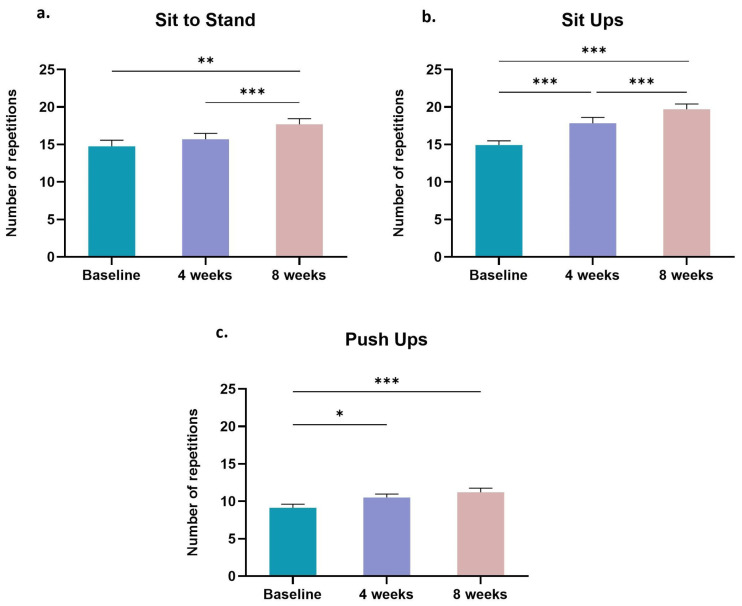
Changes in (**a**) Sit to Stand, (**b**) Sit Ups and (**c**) Push Ups performance during the tele-exercise training program in the BCa survivors. Data are presented as mean ± SE. Significantly different *: *p* < 0.05; **: *p* < 0.01; ***: *p* < 0.001.

Lower extremeties’ muscle function was improved after the completion of the eight-week tele-exercise intervention as indicated by the increase in the number of “Sit-to-Stand” repetitions (Baseline: 14.77 ± 0.81 reps vs. eight weeks: 17.69 ± 0.75 reps; *p* < 0.01; Figure 3a). Significant improvements (*p* < 0.001) were also found regarding the core muscle function as indicated by the increased number of “Sit-Ups” repetitions at the fourth (18.85 ± 0.78 reps) and eigth (19.69 ± 0.72 reps) week of the training program, respectively, compared to baseline (14.92 ± 0.56 reps) (Figure 3b). Similarly, BCa survivors’ performance in “Push-Ups” was improved gradually over the two-month training period, from 9.14 ± 0.48 reps at baseline, to 10.50 ± 0.48 reps after four weeks (*p* < 0.05) and 11.21 ± 0.55 reps after eight weeks (*p* < 0.001) of tele-exercise training (Figure 3c).

### 3.4. Quality of Life

**Figure 4:**
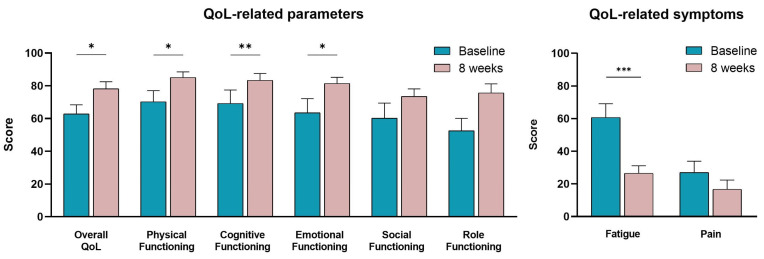
Alterations in various QoL-related parameters and symptoms assessed by the EORTC-QLQ-C30 questionnaire in women BCa survivors after their participation in an 8-week tele-exercise training program compared to baseline. Data are presented as mean ± SE. Significantly different *: *p* < 0.05; **: *p* < 0.01; ***: *p* < 0.001.

BCa survivors filled in the EORTC-QLQ-C30 questionnaire to self-evaluate various QoL-associated parameters at the beginning and the end of the tele-exercise training program (Figure 4). It was revealed that exercise had a positive impact on their overall quality of life, scoring higher their “QoL” after the completion of the exercise training (78.21 ± 4.28) compared to baseline (62.82 ± 5.56) (*p* < 0.05). Similar improvements were also self-reported regarding the scores of “Physical Functioning” (70.26 ± 6.82 vs. 85.13 ± 3.39; *p* < 0.05), “Cognitive Functioning” (69.23 ± 8.19 vs. 83.33 ± 4.22; *p* < 0.01) and “Emotional Functioning” (63.46 ± 8.73 vs. 81.41 ± 3.79; *p* < 0.05) as a result of the exercise training intervention. Social and role functioning scores exhibited a tendency to increase (improve) without reaching statistical significance (*p* > 0.05). Furthermore, regarding the long-term cancer- and treatment-related symptoms, the perceived fatigue was scored by 60.68 ± 8.40 at baseline and decreased to 26.49 ± 4.63 after the two-month exercise training (*p* < 0.001). Pain was also decreased to 16.67 ± 5.66 compared to baseline values (26.92 ± 6.94), though these changes were not statistically significant (*p* > 0.05). 

### 3.5. Post-Traumatic Stress and Anxiety

**Figure 5:**
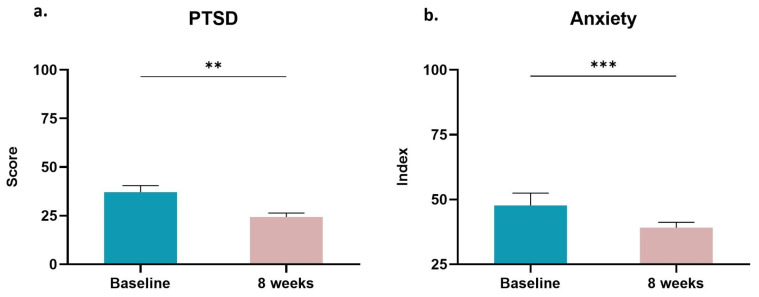
Changes in self-reported, perceived (**a**) PTSD and (**b**) anxiety in women BCa survivors after the completion of an 8-week tele-exercise training program evaluated by the specialized questionnaires “PCL-C” and “Zung Self-Rating Anxiety Scale”, respectively. Data are presented as mean ± SE. Significantly different **: *p* < 0.01; ***: *p* < 0.001.

The perceived PTSD and anxiety were also self-reported by the BCa survivors, using the specialized questionnaires “PCL-C” and “Zung Self-Rating Anxiety Scale”, respectively. At baseline, only one individual met the criteria for a full PTSD diagnosis according to DSM-IV (score: 62) in contradistinction to most of the participants (score: 37.15 ± 3.29). Overall, the PTSD symptoms were decreased after the eight-week exercise intervention (score: 24.38 ± 2.01, *p* < 0.01) even in the survivor who had a PTSD diagnosis at baseline (score: 20). Furthermore, the tele-exercise training program induced a significant reduction in the BCa survivors’ anxiety index (baseline: 47.69 ± 4.77 vs. eight weeks: 39.15 ± 2.05; *p* < 0.001; Figure 5). 

## 4. Discussion

The present study aimed at investigating the effect of a home-based supervised tele-exercise training program on physical- and mental health-associated variables in BCa survivors. Our findings suggest that a two-month tele-exercise training, consisting of aerobic, resistance and flexibility exercises, was adequate and effective in improving somatometric characteristics, physical performance, psychological parameters, such as anxiety and PTSD, and eventually overall QoL in BCa survivors. 

Specifically, the indirect indicators of body composition, BMI and waist circumference, were improved significantly after the eight-week exercise intervention. Similar results have been reported in previous remote or supervised in-person exercise programs including both aerobic and resistance training in BCa survivors [35,36]. 

Moreover, BCa survivors’ cardiorespiratory fitness was enhanced through the tele-exercise training, as it was indicated by the advances in 6MWT performance and the improvements in the perceived exertion-related variables, dyspnea and leg fatigue at the end of this submaximal exercise test. Although recent studies using home-based exercise interventions lasted 3 or 4 months and confirmed that remote exercise interventions could positively impact cardiorespiratory health of cancer survivors [22,37], to the best of our knowledge, this study is the first showing that only two months of tele-exercise training can result in significant improvements in the aerobic capacity of BCa survivors. Nevertheless, no significant changes were observed in the resting HR of the participants of the present study in response to the eight-week exercise intervention probably due to its relatively short duration. In other studies of longer duration that involved either in-person combined exercise training (aerobic and resistance) [38] or remotely performed high intensity interval training (HIIT) [37] in BCa survivors, significant changes in resting HR have been reported with this improvement being associated with reduced metabolic risk [39] and mortality [40] in this clinical population. 

Furthermore, muscle function assessment in various muscle groups showed significant improvements during the two-month exercise program in the BCa survivors. These results are in agreement with findings of previous studies where distance exercise interventions were delivered in various oncology patients either synchronously or asynchronously [41,42]. 

Engagement in physical activity programs has been suggested to improve overall functional ability and QoL and ameliorate anxiety and PTSD symptoms in cancer survivors [21,35,38,43,44]. The present study further corroborated and expanded those reports by documenting the effectiveness of a tele-exercise training intervention in improving mental health as well as overall QoL-related parameters in women BCa survivors. Furthermore, participation in this tele-exercise training program provoked positive effects regarding the perceived pain and fatigue similar to the face-to-face supervised interventions used in previous studies conducted in cancer survivors [16,45,46].

Overall, this study showed that tele-exercise was well tolerated by all the BCa survivors and their compliance to the remote exercise training program was not compromised, highlighting the feasibility of distance-supervised exercise programs in this clinical population. Moreover, the various physical and mental health benefits gained even after a shorter training period underline the efficacy and effectiveness of such programs in improving the overall QoL of BCa survivors. These findings suggest that cancer survivors should be strongly advised to participate in exercise programs, while the utilization of advanced digital technology, such as video call applications or recorded video sharing platforms, can efficiently support the access of those people to regular exercise when physical presence is not possible. However, the absence of a control group and the relatively small sample size are limitations of the present study that could lead to an underestimation of the health benefits of tele-exercise training for breast cancer survivors. Furthermore, such exercise interventions with longer durations [22,37] or combined with other health modifications like reforming patients’ dietary behaviors or implementing lifestyle counseling, [47] are expected to result in even greater improvements in health-related outcomes. 

## 5. Conclusions

The present study demonstrated that a remotely supervised exercise training program can be a feasible and effective intervention for breast cancer survivors, offering them various physical performance, mental health and QoL benefits. Nevertheless, increasing the motivation of cancer survivors to be involved in tele-exercise programs and their familiarization with video calls and other advanced technologies are emerging issues that should be addressed, in order this form of exercise intervention to be utilized for overcoming distance barriers. Moreover, this strategy could, in turn, not only increase exercise adherence but also enable a greater number of survivors to participate in physical activity programs as part of healthy lifestyle modifications for them. Furthermore, this exercise intervention could be utilized in the more aggressive phases of anti-cancer treatment when access to training facilities may be challenging, or isolation is preferred.

## Figures and Tables

**Table 1:** Characteristics of the eight-week tele-exercise training program for each of the two exercise sessions implemented within a week in the breast cancer survivors.

Total Duration: 55–60 min		
	*1st session*	*2nd session*
*5 min*	Warm up	Warm up
*15 min*	Aerobic Dance (70–80% HRmax)	Aerobic Dance (70–80% HRmax)
*~30 min*	4 sets × 10–12 reps × 4 exercises engaging major muscle groups * (45 s rest between exercises, 60 s rest between sets)	5 sets × 15 s × 4 exercises engaging major muscle groups * (25 s rest between exercises, 90 s rest between sets)
*5 min*	Breathing Exercises	Breathing Exercises
*5 min*	Cool down (flexibility exercises for all the major muscle groups)	Cool down (flexibility exercises for all the major muscle groups)

HRmax: heart rate maximum; reps: repetitions * Exercises involved legs, arms, shoulders, chest, back and trunk. The load of resistance exercises was increased at the 5th week by 15% of the initial load (at 1st week).

**Table 2:** Somatometric Characteristics of Breast Cancer survivors before and during the 8-week tele-exercise training program.

Breast Cancer Survivors	Baseline	4 Weeks	8 Weeks
Age (yrs)	58.31 ± 3.13		
Height (cm)	165.50 ± 1.48		
Body Mass (kg)	70.35 ± 2.07	70.08 ± 2.03	68.88 ± 1.89
Body Mass Index (kg/m^2^)	25.68 ± 0.62	25.58 ± 0.60	25.15 ± 0.58
Waist Circumference (cm)	96.54 ± 1.84	95.12 ± 1.91	92.31 ± 1.72

Data are presented as Mean ± SE.

## Data Availability

Further data than those already included in the article are unavailable due to privacy and ethical restrictions.
